# Data on LC–MS profile of *Brucea javanica* (L.) Merr. and the NMR data of its major indole alkaloids

**DOI:** 10.1016/j.dib.2023.109895

**Published:** 2023-12-01

**Authors:** Nor Syaidatul Akmal Mohd Yousof, Norazlan Mohmad Misnan, Akmal Hayat Abdul Karim, Murizal Zainol, Mohd Ridzuan Mohd Abd Razak, Nur Hana Md Jelas, Nor Hadiani Ismail, Adlin Afzan

**Affiliations:** a Herbal Medicine Research Centre, Institute for Medical Research, National Institutes of Health, No. 1 Jalan Setia Murni U13/52, Seksyen U13, Setia Alam, 40170 Shah Alam, Selangor Darul Ehsan, Malaysia; b Atta-ur-Rahman Institute for Natural Product Discovery, Universiti Teknologi MARA (UiTM), Puncak Alam Campus, 42300 Bandar Puncak Alam, Selangor Darul Ehsan, Malaysia; Faculty of Applied Science UiTM, 40450 Shah Alam, Selangor Darul Ehsan, Malaysia

**Keywords:** Medicinal plant, *Brucea javanica*, Chemical profiling, Dereplication, Centrifugal partition chromatography (CPC), Structural elucidation

## Abstract

This article presents two types of phytochemical data obtained from *Brucea javanica* (L.) Merr. roots, a medicinal plant belonging to the Simaroubaceae family. The high-resolution LC–MS dataset comprised the chemical profile of dichloromethane extract, which was utilised to annotate 35 chemical constituents. For annotations, the measured spectral data were compared with the *in-silico* spectral data generated from 920 molecular structures previously reported in Simaroubaceae. Indole alkaloids, quassinoids, aliphatics and lignan were the chemical groups identified in the root extract. The second dataset provides NMR spectra (^1^H, ^13^C, COSY, HMQC and HMBC) for the six indole alkaloids previously detected in LC–MS analysis and isolated through centrifugal partition chromatography. The chemical structures of all compounds were confirmed based on NMR data as bruceolline J (compound **7**), canthin-6-one-*N*-oxide (compound **10**), bruceolline E (compound **15**), 5-methoxycanthin-6-one (compound **16**), canthin-6-one (compound **20**), and 1‑hydroxy-11-methoxycanthin-6-one (compound **22**). This phytochemical data was generated to support an ongoing anti-cancer and anti-dengue study.

## Graphical abstract

**Figure:**
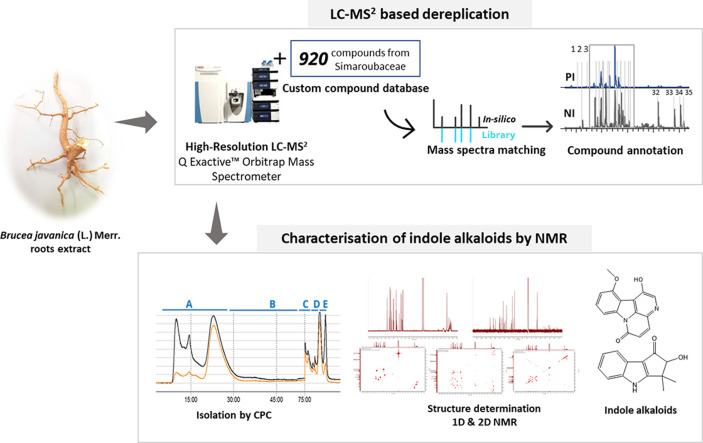

Specifications Table

**Table:** 

Subject	Chemistry
Specific subject area	Analytical Chemistry: Spectroscopy Natural products research
Type of data	Table and Figure
How the data were acquired	Liquid Chromatography-Mass Spectroscopy (LC–MS) profile of the dichloromethane (DCM) extract obtained from *B. javanica* was recorded on UPLC system coupled with Orbitrap Q Exactive™ Mass Spectrometer. A full scan with data-dependent MS^2^ (dd-MS^2^) acquisitions was applied to obtain the LC–MS data. For compound isolation, the centrifugal partition chromatography (CPC) was fitted with 250 mL column volume rotor and UV–Vis detector. The ^1^H,^13^C and 2D (COSY, HMQC, HMBC) NMR spectra for all compounds were recorded on JNM-ECZ 600 MHz NMR spectrometer.
Data format	Raw and analysed
Description of data collection	The roots of *Brucea javanica* (L.) Merr. (Simaroubaceae) were harvested from Jitra, Kedah, Malaysia at Latitude: 6°15′15.9732″ N and Longitude: 100°26′7.6848″ E. Plant authentication was performed by Forest Research Institute Malaysia (Voucher number: PID 461119-24). An LC gradient elution of 40 min total run time in positive and negative HESI modes was used to acquire two LC–MS profiles of the dichloromethane extract. The raw data was processed using Compound Discoverer™ ver. 3.1 software. The same software annotated 35 of the most intense peaks through *in-silico* spectral matching. For this purpose, a customised compound database containing 920 molecular structures was generated from the online Dictionary of Natural Products Database (Keyword: Simaroubaceae). All compounds isolated through a CPC procedure were used as reference standards to confirm the identification of 6 peaks (from the 35 peaks). The solvent system utilised for isolation consisted of hexane:ethyl acetate:methanol:water (1:2:1:2; v/v/v/v) in ascending mode. The structures of six indole alkaloids were determined based on 1D (^1^H, ^13^C) and 2D (HMBC, HMQC, COSY) NMR and by comparing the spectral data with reported values.
Data source location	Data related to plant extraction and compound isolations were generated in the Phytochemistry Laboratory, Herbal Medicine Research Centre, Institute for Medical Research (IMR), National Institutes of Health (NIH), Selangor, Malaysia. LC–MS and NMR experiments were recorded and analysed in Advanced Analytical and Biochemistry Laboratory (AABL), IMR, NIH Malaysia facilities.
Data accessibility	Repository name: Mendeley Data Direct URL to LC–MS data: https://data.mendeley.com/datasets/26v9k5t2zx Direct URL to NMR data: https://data.mendeley.com/datasets/pfgpvykyym

## 1 Value of the Data

• This dataset provides a comprehensive LC–MS chemical profile, compound annotations and characterisations of the isolated indole alkaloids from the roots extract of *B. javanica*, a less studied plant part.
• In order to dereplicate the chemical constituents, ethnobotanists, chemists and natural product researchers investigating the medicinal properties of this plant can benefit from the LC–MS profiling and annotation data.
• The reported chemical constituents could be a valuable resource for chemical marker selection during the development of standardised extract and quality control.
• The NMR spectral data provide updated references and spectra for indole alkaloids, which is useful in elucidating structurally related indole alkaloids.

## 2 Objective

*B. javanica* has been widely studied for various bioactivities including anti-cancer, anti-plasmodial, anti-inflammatory, and anti-viral [1], [2], [3], [4]. While many phytochemical data described the seed extract [5,6], this article aimed to provide comprehensive and updated references for the chemical constituents in root extract.

## 3 Data Description

**Fig. 1:**
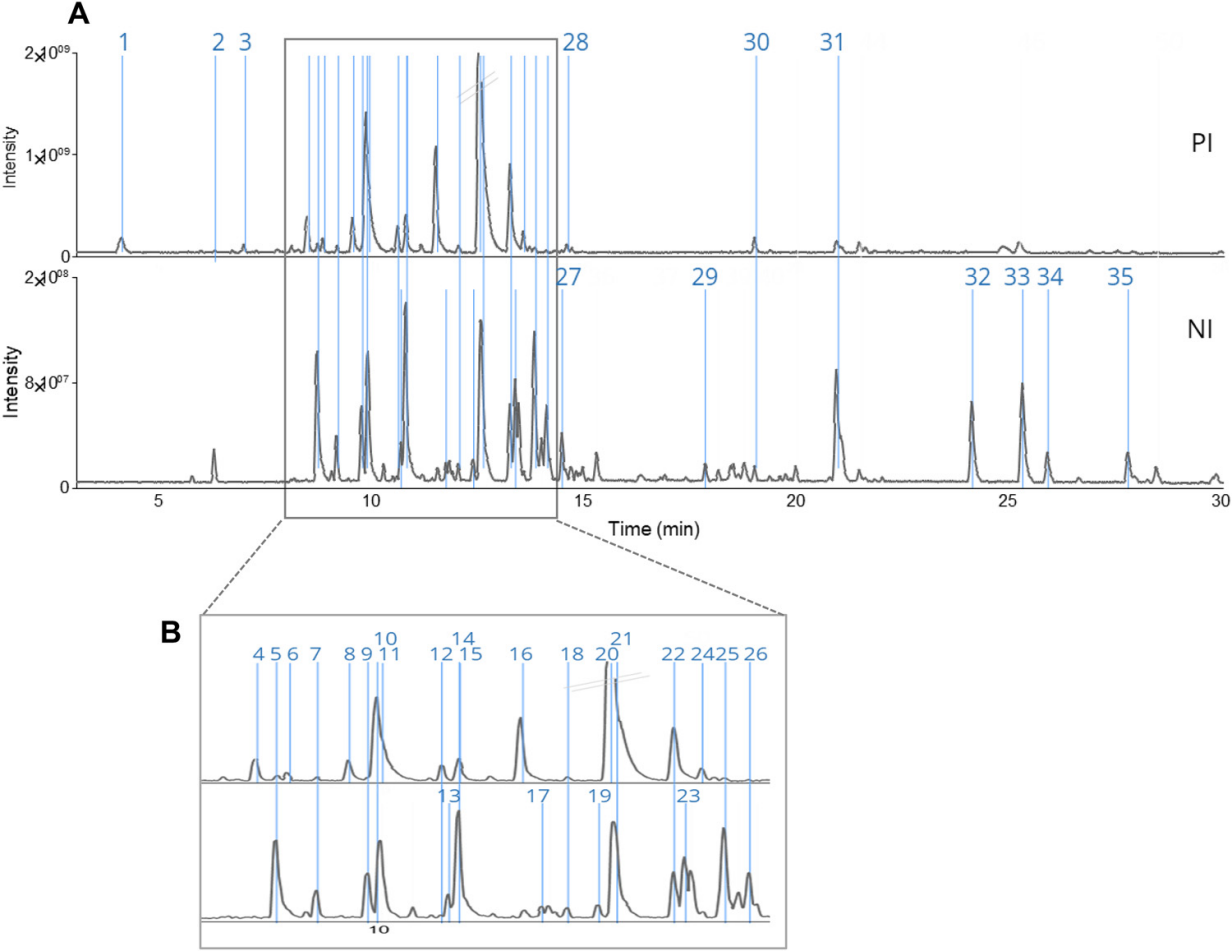
(**A**) PI and NI base peak chromatograms obtained from LC–MS analysis of *B. javanica* roots extract and **B**) the zoom-in chromatograms at 8–14 min. The numbered peaks are corresponding to Table 1. All unlabelled peaks are unknown.

The LC–MS data for *B. javanica* dichloromethane (DCM) extract consists of the base peak chromatograms in positive (PI) and negative ionisation (NI) modes (Fig. 1). The raw files are deposited in Mendeley Data: https://data.mendeley.com/datasets/26v9k5t2zx. Table 1 describes the MS^1^ and MS^2^ data for 35 chemical constituents, annotated by *in-silico* spectral matching. For this purpose, the Simaroubaceae database containing 920 molecular structures was retrieved from online Dictionary of Natural Products [7]. From the 35 compounds, 12 were detected solely in PI, 10 in NI, and 13 in both PI and NI modes. The two dominant chemical families present in the extract were indole alkaloids (51 %) and quassinoids (31 %), while others were lignans and aliphatics (17 %).

**Table 1:** Metabolite annotations of indole alkaloids, quassinoids, lignans and aliphatics in DCM extract of *B. javanica* roots by LC–MS analysis.

Peak No.	Ionisation	Measured *m/z*	Adduct	RT (min)	Chemical class	Molecular formula	Tentative identification	MS^2^
1	PI	218.1170	[M+H]^+^	4.03	Indole alkaloids	C_13_H_15_NO_2_	1-(1H-Indol-3-yl)−3-methyl-2-buten-1-one; 2,3-Dihydro, 2R‑hydroxy	200.11, 190.95, 170.1, 157.09, 130.06
2	PI	411.1642	[M+H]^+^	6.24	Quassinoids	C_20_H_26_O_9_	Brucein D	393.16, 375.14, 271.13, 225.13, 211.11, 69.03
3	PI	199.0862	[M+H]^+^	6.93	Indole alkaloids	C_12_H_10_N_2_O	1-Hydroxymethyl-β-carboline	181.08, 154.06, 128.95, 88.97, 55.93
4	PI	197.1069	[M+H]^+^	8.43	Indole alkaloids	C_13_H_12_N_2_	1-Ethyl-β-carboline	182.08, 154.53, 143.02, 113.96, 106.99
5	PI	485.1644	[M+H]^+^	8.67	Quassinoids	C_22_H_28_O_12_	Bruceaketolic acid	425.15, 407.13, 379.14, 213.09, 139.04
	NI	483.1510	[M-H]^−^					
6	PI	255.1122	[M+H]^+^	8.80	Indole alkaloids	C_15_H_14_N_2_O_2_	4,7-Dimethoxy-1-vinyl-β-carboline	223.09, 195.09, 181.08, 163.06, 81.77, 51.36
7	PINI	216.1015	[M+H]^+^	9.13	Indole alkaloids	C_13_H_13_NO_2_	Bruceolline J (Fig. 3)	198.09, 170.1, 155.07, 143.09, 91.96, 80.17, 61.21
	NI	214.0872	[M-H]^−^					
8	PI	237.0653	[M+H]^+^	9.48	Indole alkaloids	C_14_H_8_N_2_O_2_	Canthin-2,6-dione	181.08, 160.67, 93.38, 81.53, 56.56
9	PI	479.1538	[M+H]^+^	9.71	Indole alkaloids	C_23_H_26_O_11_	Dehydrobrucein B	220.06, 192.07, 151.95, 121.49, 88.95, 53.04
	NI	477.1406	[M-H]^−^					
10	PI	237.0652	[M+H]^+^	9.82	Indole alkaloids	C_14_H_8_N_2_O_2_	Canthin-6-one-*N*-oxide (Fig. 4)	419.13, 384.89, 297.11, 265.09, 203.11, 185.09
	NI	235.0512	[M-H]^−^					
11	PI	227.0810	[M+H]^+^	9.86	Indole alkaloids	C_13_H_10_N_2_O_2_	1-Methoxycarbonyl-β-carboline	213.07, 195.06, 185.07, 167.06, 140.05, 82.28
12	PI	271.107	[M+H]^+^	10.55	Indole alkaloids	C_15_H_14_N_2_O_3_	7-Methoxy-β-carboline-1-propanoic acid	239.08, 195.09, 185.07, 167.07, 141.76, 115.05
	NI	269.0928	[M-H]^−^					
13	NI	567.2086	[M-H]^−^	10.64	Quassinoids	C_27_H_36_O_13_	Javanic acid B	331.12, 249.1, 221.29, 172.99, 143.07, 99.08, 57.03
14	PI	237.0649	[M+H]^+^	10.75	Indole alkaloids	C_14_H_8_N_2_O_2_	5-Hydroxycanthin-6-one	209.0709, 193.0754, 181.0759, 155.0598, 128.0493, 84.9685, 61.8951
15	PI	214.0858	[M+H]^+^	10.75	Indole alkaloids	C_13_H_11_NO_2_	Bruceolline E (Fig. 5)	186.09, 168.08, 158.1, 144.08, 93.36, 61.16
	NI	212.0715	[M-H]^−^					
16	PI	251.0809	[M+H]^+^	11.46	Indole alkaloids	C_15_H_10_N_2_O_2_	5-Methoxycanthin-6-one (Fig. 6)	236.06, 223.09, 208.06, 180.07, 160.07, 99.19, 89.18, 64.85
17	NI	609.2194	[M-H]^−^	11.71	Quassinoids	C_29_H_38_O_14_	Bruceanic acid C	312.44, 155, 143.07, 111.01, 99.08, 83.01, 57.03
18	PI	357.1325	[M+H]^+^	11.99	Lignans	C_20_H_20_O_6_	4,7′-Epoxy-4′,9′-dihydroxy-3′,5-dimethoxy-3,8′-lign-7-en-9-al	339.12, 321.11, 307.1, 279.1, 261.09, 165.05, 137.06, 55.02
	NI	355.1191	[M-H]^−^					
19	NI	545.1667	[M-H]-	12.35	Quassinoids	C_27_H_30_O_12_	Bruceanic acid B	291.59, 261.09, 187.67, 121.03, 101.8, 59.01
20	PI	221.0703	[M+H]^+^	12.47	Indole alkaloids	C_14_H_8_N_2O_	Canthin-6-one (Fig. 7)	193.09, 166.07, 119.46, 100.99, 50.35
21	PI	527.2110	[M+H]^+^	12.55	Quassinoids	C_25_H_34_O_12_	Samaderine A	389.12, 361.13, 285.11, 243.1, 213.09, 139.04, 85.07, 57.07
	NI	525.1979	[M-H]^−^					
22	PI	267.0756	[M+H]^+^	13.20	Indole alkaloids	C_15_H_10_N_2_O_3_	1-Hydroxy-11-methoxycanthin-6-one (Fig. 8)	252.05, 224.06, 138.13, 117.31, 88.99, 50.86
	NI	265.0613	[M-H]^−^					
23	NI	609.2191	[M-H]^−^	13.33	Quassinoids	C_29_H_38_O_14_	Bruceanic acid C	414.43, 240.8, 176.42, 154.88, 125.06, 97.06, 59.01
24	PI	251.0809	[M+H]^+^	13.51	Indole alkaloids	C_15_H_10_N_2_O_2_	1-Methoxycanthin-6-one	236.06, 222.39, 202.09, 126.45, 119.46, 75.36, 50.19
25	PI	523.2166	[M+H]^+^	13.78	Quassinoids	C_26_H_34_O_11_	Bruceine A	439.16, 403.14, 365.2, 299.13, 267.1, 201.09, 85.06, 57.07
	NI	567.2084	[M+FA-H]^−^					
26	PI	553.2270	[M+H]^+^	14.04	Quassinoids	C_27_H_36_O_12_	Bruceanic acid A	407.13, 379.14, 285.11, 243.1, 111.08, 93.07, 55.05
	NI	551.2136	[M-H]^−^					
27	NI	651.2297	[M-H]^−^	14.43	Quassinoids	C_31_H_40_O_15_	Javanicoside G	425.6, 291.03, 254.47, 167.07, 125.06, 111.01, 59.01
28	PI	251.0810	[M+H]^+^	14.54	Indole alkaloids	C_15_H_10_N_2_O_2_	Picrasidine L	236.06, 208.06, 173.17, 130.57, 80.49, 64.43, 59.44
29	NI	212.0716	[M-H]^−^	17.80	Indole alkaloids	C_13_H_11_NO_2_	3,4-Dihydro-3,3-dimethylcyclopent[*b*]indole-1,2-dione	197.05, 184.08, 169.05, 156.08, 134.95, 92.2, 62.66
30	PI	369.1955	[M+H]^+^	18.96	Quassinoids	C_19_H_28_O_7_	1,4-Dehydrocedronolactone A	354.17, 298.15, 283.12, 258.19, 160.28, 74.42, 60.42
	NI	367.1819	[M-H]^−^					
31	PI	279.2312	[M+H]^+^	20.89	Aliphatic	C_18_H_30_O_2_	17-Octadecen-6-ynoic acid	261.22, 243.21, 173.13, 123.12, 109.1, 95.09, 81.07, 67.05
	NI	295.2276	[M+H2O]^−^					
32	NI	295.2277	[M-H]-	24.09	Aliphatic	C_18_H_32_O_3_	8-Oxo-octadec-9-enoic acid	249.22, 196.02, 155.14, 141.13, 127.11, 97.87, 79.17
33	NI	271.2276	[M-H]-	25.27	Aliphatic	C_16_H_32_O_3_	8-Hydroxyhexadecanoic acid; (±)-form	253.22, 225.22, 158.98, 125.93, 90.53, 69.82, 64.59
34	NI	297.2436	[M-H]-	25.86	Aliphatic	C_18_H_34_O_3_	7-Oxooctadecanoate	251.24, 222.04, 195.25, 73.52, 65.53, 59.39
35	NI	299.2592	[M-H]-	27.75	Aliphatic	C_18_H_3_6O_3_	2-Hydroxystearate	288.6, 274.16, 253.25, 158.51, 125.06, 90.77, 80.22, 61.55

*Note:* For all peaks, the mass error set for the molecular formula was 5 ppm, and the FISh Coverage score for spectral matching was mostly ca. 40%.

**Fig. 2:**
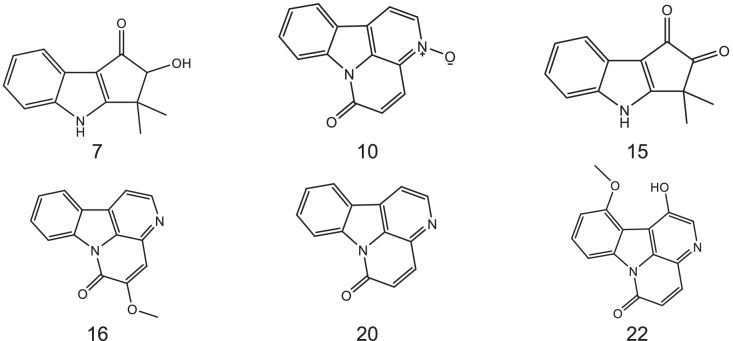
Indole alkaloids structures.

**Fig. 3:**
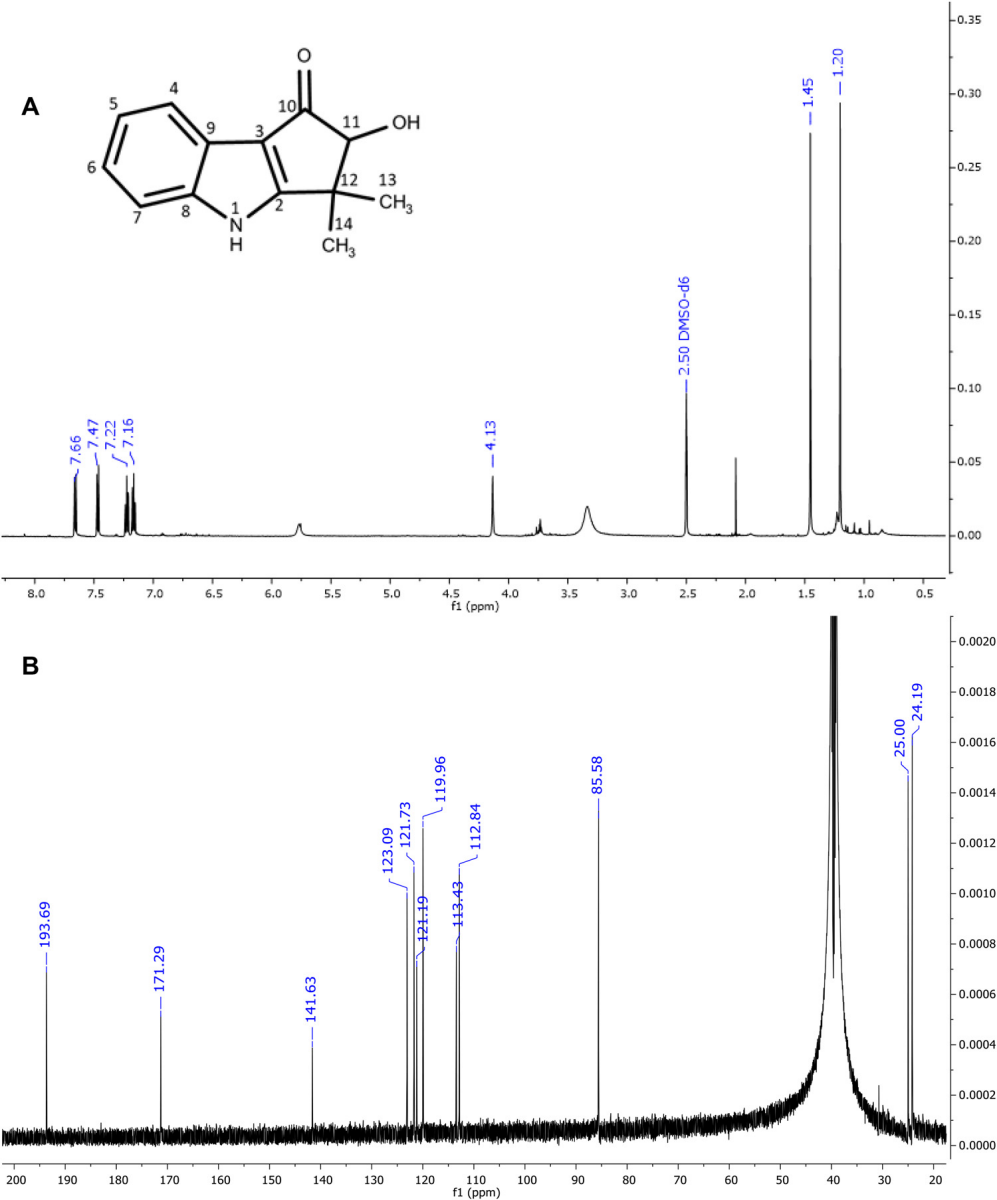
**(A)**^1^H and **(B)**^13^CNMR spectra of bruceolline J (**7**).

**Fig. 4:**
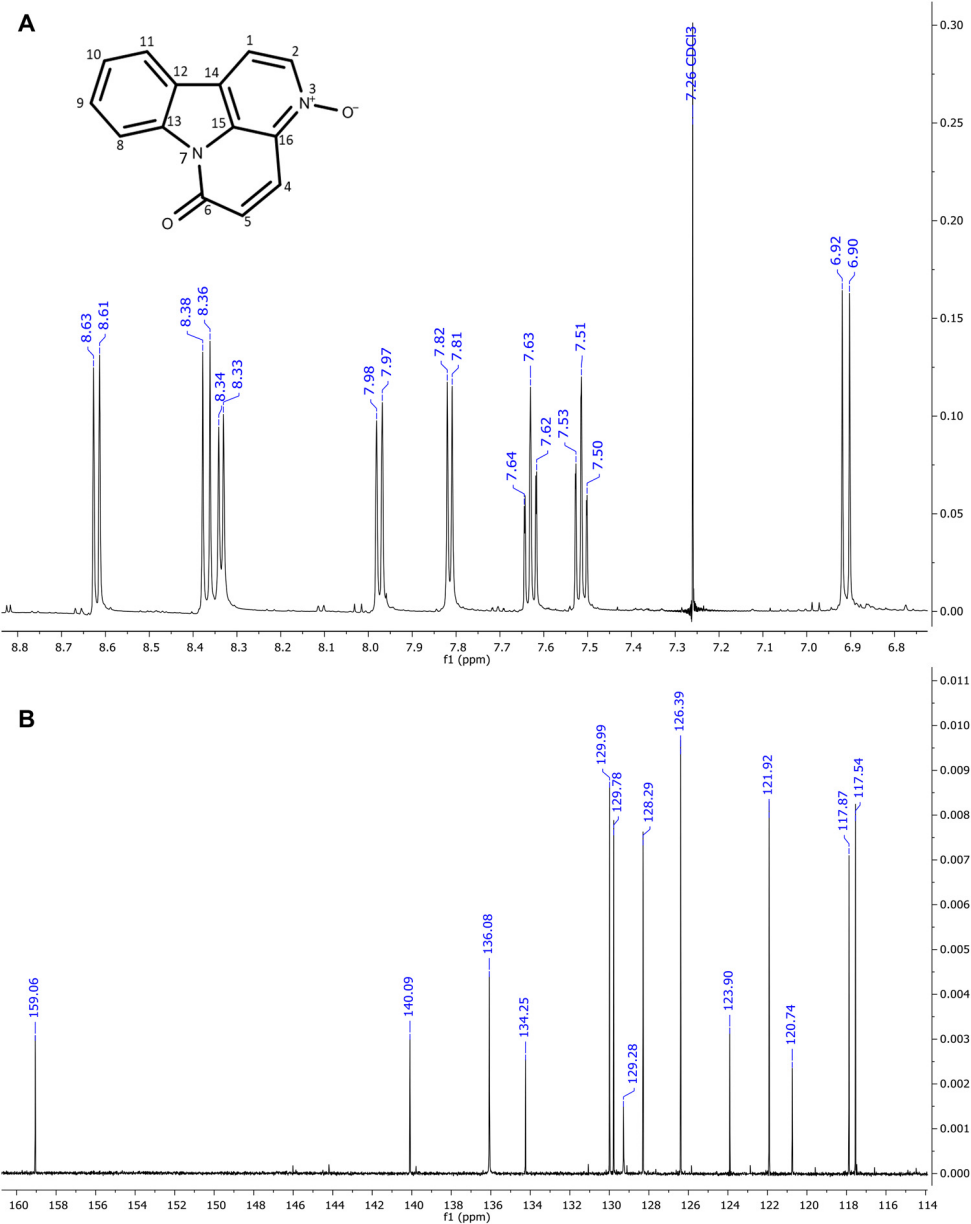
(**A**) ^1^H and (**B**) ^13^C NMR of canthin-6-one-*N*-oxide (**10**).

**Fig. 5:**
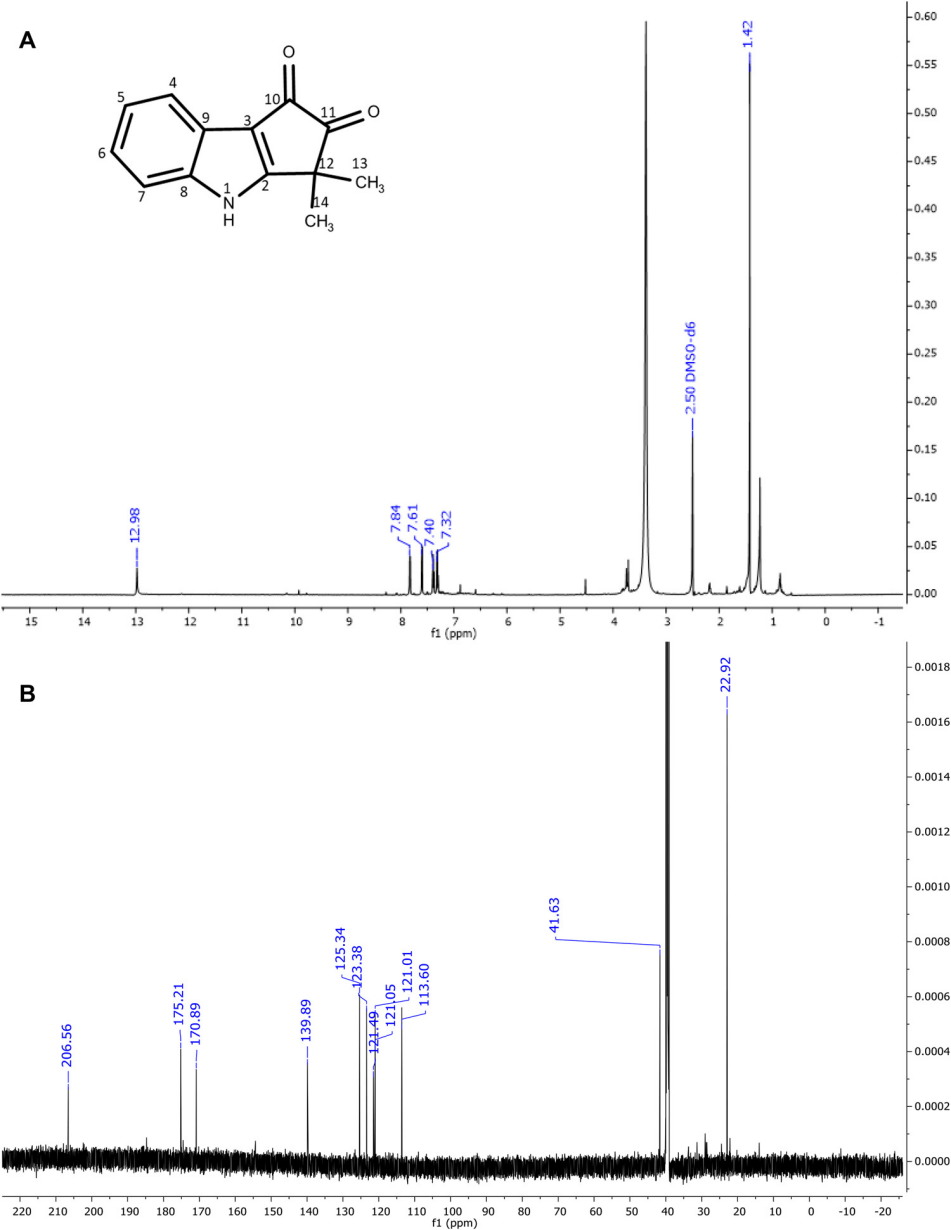
(**A**) ^1^H and (**B**) ^13^C NMR of bruceolline E (**15**).

**Fig. 6:**
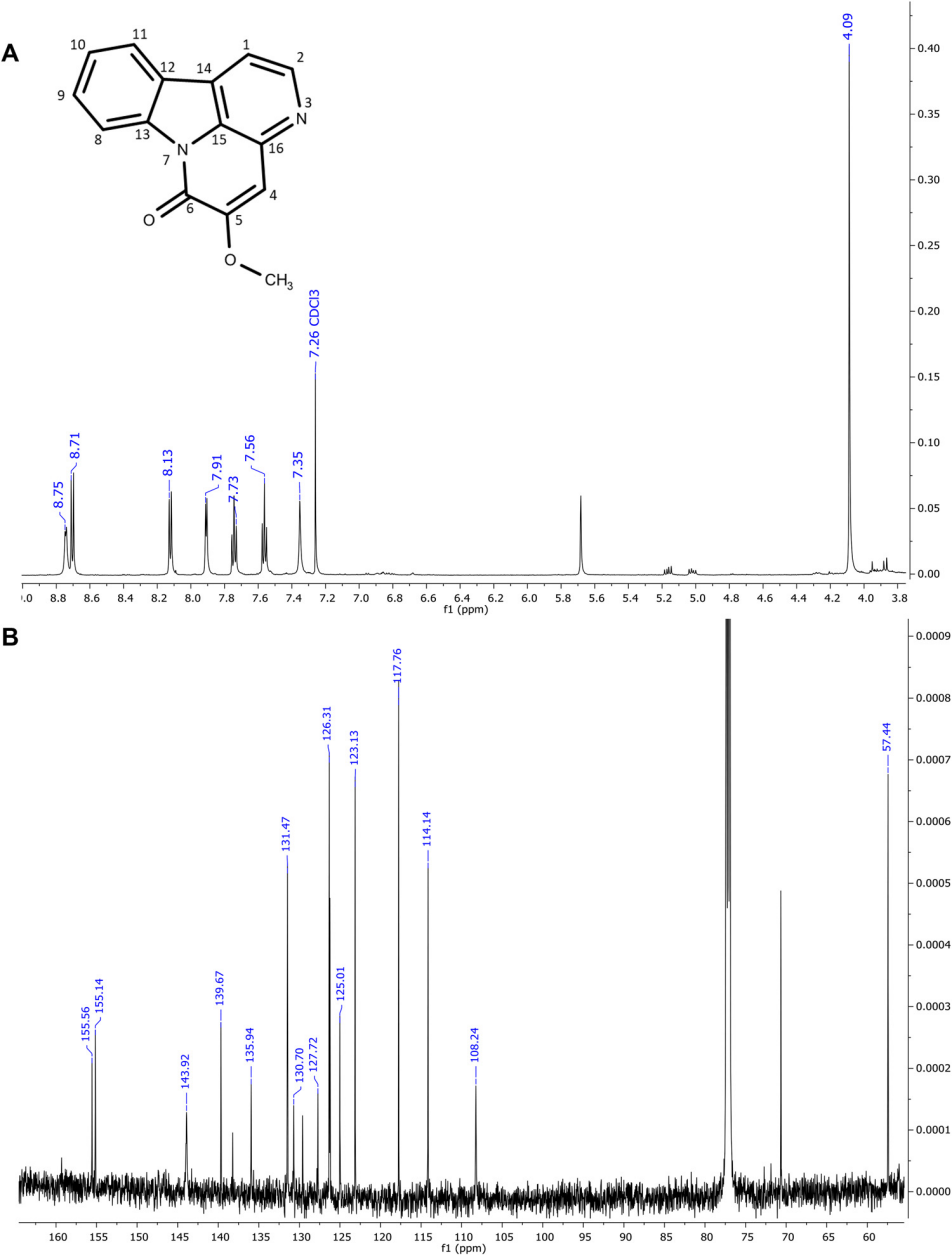
(**A**) ^1^H and (**B**) ^13^C NMR of 5-methoxycanthin-6-one (**16**).

**Fig. 7:**
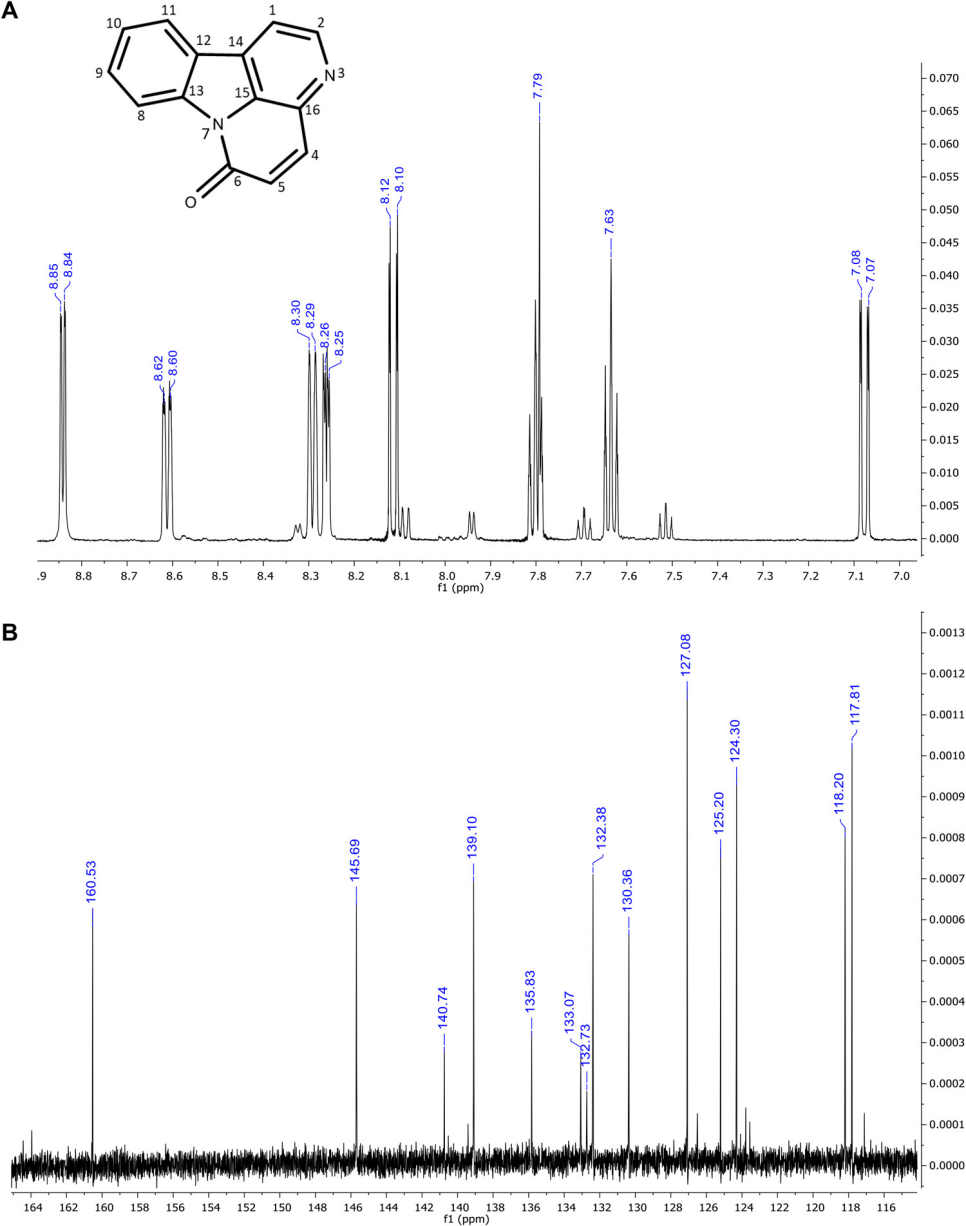
(**A**) ^1^H and (**B**) ^13^C NMR of canthin-6-one (**20**).

**Fig. 8:**
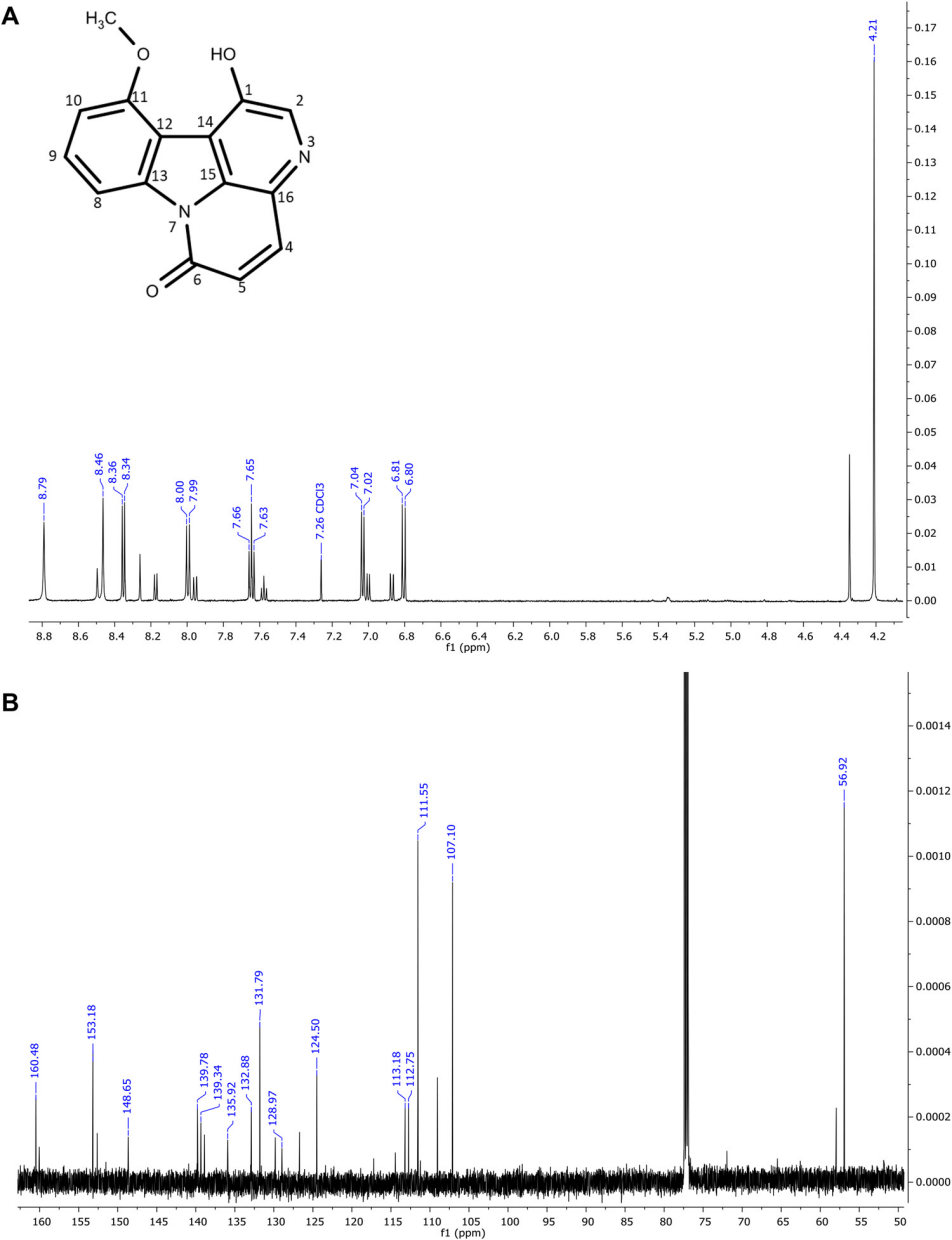
(**A**) ^1^H and (**B**) ^13^C NMR of 1‑hydroxy-11-methoxycanthin-6-one (**22**).

The six major constituents (Fig. 2) detected in the LC-MS were isolated from DCM extract using CPC and used as reference standards to confirm the chemical identifications. All isolates were characterised as indole alkaloids based on the ^1^H NMR (Table 2) and ^13^C NMR (Table 3) assignments. The ^1^H, ^13^C spectra and chemical structures are included as follows: bruceolline J (**7**) (Fig. 3); canthin-6-one-*N*-oxide (**10**) (Fig. 4); bruceolline E (**15**) (Fig. 5); 5-methoxycanthin-6-one (**16**) (Fig. 6); canthin-6-one (**20**) (Fig. 7); 1‑hydroxy-11-methoxycanthin-6-one (**22**) (Fig. 8). The 2D NMR data (COSY, HMQC, HMBC) can be accessed here: https://data.mendeley.com/datasets/pfgpvykyym.

**Table 2:** ^1^H NMR spectroscopic data of all isolated compounds (**7, 10, 15, 16, 20** and **22**).

^1^H positions	7	10	15	16	20	22
1	-	7.81, d (6.6)	12.98, s [-NH]	7.91, d (5.0)	8.26, d (5.1)	8.79, s [-OH]
2	-	9.34, d (6.6)	-	8.74, d (5.3)	8.84, d (5.2)	8.46, s
3	-	-	-	-	-	-
4	7.66, d (7.7)	8.37, d (10)	7.83, d (7.7)	7.35, s	8.11, d (9.8)	8.00, d (9.7)
5	7.16, td (7.6)	6.91, d (10)	7.80, dd (7.32)	–	7.08, d (9.8)	6.81, d (9.7)
6	7.25–7.20, m	–	7.39, t (7.7)	–	–	–
7	7.47, d (8.1)	–	7.60, d (7.8)	–	–	–
8	-	8.62, d (8.2)	-	8.70, d (8.2)	8.61, d (8.2)	9.35, d (8.2)
9	-	7.63, t (8.4)	-	7.74, t (7.8)	7.81–7.79, m	7.65, t (8.2)
10	-	7.51, t (7.6)	-	7.56, t (7.6)	7.65–7.62, m	7.03, d (8.2)
11	4.13, s	7.97, d (7.8)	-	8.12, d (7.6)	8.29, d (7.8)	–
12	-	–	-	–	–	–
13	1.45, s [—CH_3_]	–	1.42, s [—CH_3_]	–	–	–
14	1.20, s [—CH_3_]	–	1.42, s [—CH_3_]	–	–	–
5-OCH_3_	–	–	–	4.09, s	–	–
11-OCH_3_	–	–	–	–	–	4.21, s

*Notes:* Chemical shifts are in ppm. The coupling constant reported in brackets is in Hz. All protons correspond to methine except those reported in square brackets. Samples were prepared in these solvents: methanol-d4 (Compound **20**), chloroform-d3 (compounds **10, 16,** and **22**) and dimethylsulfoxide-d6 (compounds **7** and **15**).

**Table 3:** ^13^C NMR spectroscopic data of all isolated compounds (**7, 10, 15, 16, 20** and **22**).

^13^C positions	7	10	15	16	20	22
1	–	117.87 CH	–	114.14 CH	118.20 CH	148.65 COH
2	171.29 C	136.08 CH	170.89 C	143.92 CH	145.99 CH	135.92 CH
3	113.43 C	–	121.05 C	–	–	–
4	119.96 CH	129.78 CH	121.01 CH	108.24 CH	139.10 CH	139.34 CH
5	121.73 CH	128.29 CH	123.88 CH	155.56 CO	130.36 CH	124.50 CH
6	123.09 CH	159.06 CO	125.34 CH	155.14 CO	160.53 CO	160.48 CO
7	112.84 CH	–	113.60 CH	–	–	–
8	141.63 C	117.54 CH	139.89 C	117.76 CH	117.81 CH	111.55 CH
9	121.19 C	129.99 CH	1241.49 C	131.47 CH	132.38 CH	131.9 CH
10	193.69 CO	126.39 CH	175.21 CO	126.31 CH	127.08 CH	107.10 CH
11	85.58 COH	121.92 CH	206.56 CO	123.13 CH	124.30 CH	153.18 CO
12	39.91 C	123.90 C	41.63 C	139.67 C	125.20 C	113.18 C
13	25.00 CH_3_	140.99 C	22.92 CH_3_	130.7 C	140.74 C	139.78 C
14	24.19 CH_3_	120374 C	22.92 CH_3_	125.01 C	132.73 C	112.75 C
15	–	134.25 C	–	135.94 C	133.07 C	132.88 C
16	–	129.28 C	–	127.72 C	135.83 C	128.97 C
5-OCH_3_	–	–	–	57.44	–	–
11-OCH_3_	–	–	–	–	–	56.92

## 4 Experimental Design, Materials and Methods

### 4.1 Plant collection, authentication, and extraction

The roots of *B. javanica,* known in Malay as “melada pahit” were harvested in August 2019 from Jitra, Kedah, Malaysia. The plant was authenticated by a botanist from Forest Research Institute Malaysia (FRIM) as *Brucea javanica* (L.) Merr (Voucher number: PID 461119-24). The dried roots were ground into powder (1 kg), defatted with hexane and extracted with DCM successively for 24 hr each. This step was repeated 3 times with fresh solvent with an approximately 8 L solvent used. The extract yield was 4.68 g (0.50 % yield, wt/wt).

### 4.2 Chemical profiling of Brucea javanica roots extract

Chemical profiling of the DCM extract was obtained from LC–MS. Briefly, 1 µL of the crude extract (5 mg/mL in methanol) was flowed (0.3 mL/min) through ACQUITY UPLC ® HSS T3 1.8 µm, 2.1 mm X 100 mm column (Waters Corporation, Ireland). Throughout analysis, column and sample compartment temperatures were maintained at 40 °C and 10 °C, respectively. The mobile phase comprised 0.1 % formic acid in water (A) and 0.1 % formic acid in acetonitrile (B). The LC gradient was as follows: 5 % B for 2 min; 5 % B – 100 % B from 2- 30 min; 100 % B from 30 to 35 min; 100 % B to 5 % B from 35 to 35.5 min; and finally, 4.5 min column equilibration with 5 % B.

The high-resolution mass spectrometer was operated at 70,000 orbitrap resolutions with a maximum IT of 250 ms for full scan. Data-dependent MS^2^ (dd-MS^2^) events were performed on the top five most abundant ions detected in the MS full-scan. The MS^2^ data was obtained at 17,000 resolutions with maximum IT of 60 ms. The normalised stepped collision energy (NCE) was set to 15, 30, and 45 V. Positive and negative ionisation modes were applied to acquire a comprehensive list of chemical compounds. The optimised HESI parameters were set as follows: spray voltage, 4.0 kV (PI) and 3.5 kV (NI); capillary temperature, 350 °C; auxiliary gas flow rate, 11 L/min; sheath gas flow, 4 kV; S-lens RF level, 60 %. The mass range was obtained from *m/z* 100 to 1500.

### 4.3 LC–MS data acquisition and metabolite annotation

The raw scans of Q Exactive™ Orbitrap Mass Spectrometer data were imported into Compound Discoverer™ 3.1 software (CD) (Thermo Fisher Scientific, Waltham, MA, USA) for pre-processing. The parameters for spectra selection, alignment of the retention time nodes, compound detections, compound grouping to specify the preferred adducts and blank removal were optimised accordingly. *B. javanica* metabolite annotation was predefined using “Untargeted Metabolomics with Statistics Detect Unknowns with ID using Online Databases and mzLogic” workflow template. A customised database containing 920 compounds previously reported from Simaroubaceae family was retrieved from Dictionary of Natural Product (DNP) and loaded into CD as “mass lists”. The mass lists include chemical names, molecular formulas, molecular weights, and chemical structures.

### 4.4 Centrifugal partition chromatography isolation procedure

Indole alkaloids isolation was performed using a centrifugal partition chromatography CPC-250 system (Armen Instrument, Saint-Ave, France) fitted with a 250 mL hydrostatic column and UV/VIS detector. A ternary biphasic system consisting of a mixture of hexane:ethyl acetate:methanol:water, 1:2:1:2 (Arizona K) was prepared to obtain two immiscible liquid phases; upper and lower phases. By selecting ascending as the elution mode, the column was loaded with the aqueous stationary phase (lower phase) at a flow rate of 30 mL/min with 600 rpm rotation speed for 10 min. Subsequently, the organic mobile phase (upper phase) was pumped into the column at 10 mL/min with 1500 rpm rotation speed for 20 min to achieve column equilibration. The DCM extract (83 mg/mL in a mixture of upper and lower phase (5:1, *v:v*)) was then injected through the injection loop and monitored at UV 254 and 320 nm. During the 90 min elution, fractions were collected in every 10 mL/ tube. All 65 fractions were screened by HPTLC and fractions containing the same component were combined to afford 5 fractions (FrA, FrB, FrC, FrD and FrE). Further purification of these fractions was performed using JAI LC–200NEXT preparative HPLC system (Japan Analytical Industry, Co., Ltd, Meguro Tokyo, Japan). The purification of fraction FrA on a silica column (JAIGEL-SIL, SH-043–15 (150 × 21.2 mm)) and an isocratic solvent system (DCM:methanol (97:3)) yielded compounds **7** and **10**. For FrC and FrD purification, isocratic mobile phase of DCM:methanol (98:2) was applied. This purification yielded compounds **15, 16** and **20, 22,** respectively.

### 4.5 Characterisation of indole alkaloids by NMR

The one-dimensional (^1^H, ^13^C) and two-dimensional (COSY, HMQC, HMBC) NMR analyses were recorded on a JNM-ECZ 600 MHz NMR spectrometer (JEOL Ltd., Tokyo, Japan) system. This system is equipped with a 5-mm digital auto-tune Royal probe in a variable temperature (VT) at 298 K and controlled by JEOL Delta NMR Software. ^1^H NMR analysis was carried out with 16 scans, 5 s delay, spectral width −2.0 to 15 ppm, spectral resolution 0.5496 Hz, data point 16,384, and receiver gains at 50. The ^13^C NMR analysis was carried out with more than 100,00 scans (depending on samples), 2 s delay, 1.1514 Hz spectral resolution, and 32,768 data point. The receiver gain was adjusted automatically for each sample before acquisition to avoid receiver overload. The default JEOL pulse sequence methods were used for all 2D NMR experiments. Data for ^1^H NMR are reported as follows: chemical shift (δ ppm), multiplicity (singlet (s), doublet (d), triplet (t), quartet (q), quintet (p), multiplet (m), doublet of doublets (dd), doublet of triplets (dt), broad (br)), and coupling constant (*J* in Hz). The chemical shift for ^13^C NMR data is reported in ppm. All spectra were referenced using solvent peak. The NMR data were processed in MestReNova *ver.* 14 software (Mestrelab Research, SL, Spain). The structures of all compounds were determined based on ^1^H, ^13^C NMR, HMQC, HMBC, and COSY data. In addition, the proposed structures were verified by two approaches: comparison with reported values [1,[8], [9], [10], [11], [12], [13]] and verification with >95 % confidence using ACD/Labs NMR Structure elucidator (ACD/Labs, Ontario, Canada).

## Ethics Statements

This research does not involve animal or human samples and therefore requires no ethical approval.

## CRediT authorship contribution statement

**Nor Syaidatul Akmal Mohd Yousof:** Formal analysis, Investigation, Writing – original draft, Visualization, Data curation. **Norazlan Mohmad Misnan:** Investigation, Validation, Writing – original draft, Visualization, Data curation. **Akmal Hayat Abdul Karim:** Investigation, Resources. **Murizal Zainol:** Funding acquisition, Resources, Project administration. **Mohd Ridzuan Mohd Abd Razak:** Resources. **Nur Hana Md Jelas:** Resources. **Nor Hadiani Ismail:** Supervision. **Adlin Afzan:** Conceptualization, Methodology, Resources, Data curation, Formal analysis, Writing – review & editing, Supervision, Project administration.

## Data Availability

LC-MS profile dataset for Brucea javanica extract (Original data) (Mendeley Data) NMR dataset for indole alkaloids isolated from Brucea javanica extract (Original data) (Mendeley Data)
